# Proline Improves Pullulan Biosynthesis Under High Sugar Stress Condition

**DOI:** 10.3390/microorganisms12122657

**Published:** 2024-12-21

**Authors:** Keyi Liu, Junqing Wang, Feng Li, Ruiming Wang, Qingming Zeng, Zhenxing Zhang, Hongwei Liu, Piwu Li

**Affiliations:** 1 School of Bioengineering, Qilu University of Technology (Shandong Academy of Sciences), Jinan 250353, China; lky6688qlu@163.com (K.L.); wjqtt.6082@163.com (J.W.); ruiming3k@163.com (R.W.); liu_hongwei0310@163.com (H.L.); 2 State Key Laboratory of Biobased Material and Green Papermaking, Qilu University of Technology (Shandong Academy of Sciences), Jinan 250353, China; 3 Shandong Shendong Intelligent Equipment Co., Ltd., Dezhou 253000, China; lf1769@163.com (F.L.); sales06@ndlcraft.com (Z.Z.); 4 Shandong Mimei Biological Technology Co., Ltd., Weifang 262600, China; chinabld@126.com

**Keywords:** *Aureobasidium pullulans*, pullulan, proline, hyperglycemia, hypertonicity, RNA sequencing

## Abstract

Pullulan is an extracellular polysaccharide produced via the fermentation of *Aureobasidium pullulans*. However, high sugar concentrations and hyperosmotic stress limit pullulan biosynthesis during the fermentation process. Therefore, we investigated the effects of proline supplementation on *A*. *pullulans* growth and pullulan biosynthesis under high sugar and hyperosmotic stress using physiological, biochemical, and transcriptomic analyses. High sugar concentrations significantly inhibited *A*. *pullulans* growth and pullulan biosynthesis. High sugar and hyperosmotic stress conditions significantly increased intracellular proline content in *A*. *pullulans*. However, treatment with proline (400 mg/L proline) significantly increased biomass and pullulan yield by 10.75% and 30.06% (174.8 g/L), respectively, compared with those in the control group. To further investigate the effect of proline on the fermentation process, we performed scanning electron microscopy and examined the activities of key fermentation enzymes. Proline treatment preserved cell integrity and upregulated the activities of key enzymes involved in pullulan biosynthesis. Transcriptome analysis revealed that most differentially expressed genes in the proline group were associated with metabolic pathways, including glycolysis/gluconeogenesis, pyruvate metabolism, and sulfur metabolism. Conclusively, proline supplementation protects *A*. *pullulans* against high sugar and hyperosmotic stress, providing a new theoretical basis and strategy for the efficient industrial production of pullulans.

## 1. Introduction

Pullulan is an extracellular polysaccharide produced during the fermentation of *Aureobasidium pullulans*, which is widely utilized in the food, medical, and chemical industries [1,2,3,4]. However, high production costs significantly restrict its application in various sectors. Owing to the continuous increase in the demand for pullulan, the development of cost-effective production methods has attracted considerable attention [5,6]. Currently, pullulan production predominantly involves a complex process known as fed-batch fermentation. However, strategies to improve *A*. *pullulans* adaptation to high sugar conditions may simplify the fermentation steps, resulting in decreased fermentation time and increased production efficiency [7]. Moreover, maintaining a high carbon-to-nitrogen ratio during fermentation is essential to promote carbon flux toward the accumulation of extracellular polysaccharides [8,9]. Consequently, increasing the sugar concentration of the fermentation medium is necessary to achieve improved pullulan yield. However, the strong osmotic pressure resulting from elevated sugar concentrations can inhibit *A*. *pullulans* growth [10,11,12]. Notably, environmental stress exceeding certain thresholds can inhibit microbial growth and metabolism, thereby limiting fermentation and pullulan production [13]. Therefore, identifying strategies to mitigate high sugar and hyperosmotic stress is critical for increasing pullulan yield.

Microorganisms typically accumulate compatible solutes in response to hyperosmotic stress. Amino acids and their derivatives are widely recognized as compatible solutes that confer intracellular resistance to environmental stresses. Importantly, glycine, proline, and glutamic acid are commonly used protectants against osmotic stress. Protective agents are frequently employed in hypertonic fermentation systems because they can accumulate in significant quantities within the cells of various microorganisms without adversely affecting their viability [14,15,16]. However, the effect of osmoprotectants on hyperosmotic stress responses in *A*. *pullulans* remains unclear.

Therefore, this study aimed to investigate the effects of the osmoprotectant proline on *A*. *pullulans* growth and pullulan biosynthesis under high sugar and hyperosmotic conditions. Specifically, we investigated whether exogenous supplementation with proline can enhance the ability of *A*. *pullulans* to withstand high sugar and hyperosmotic conditions. This study’s findings reveal that *A. pullulans* accumulates proline in small amounts during growth under these conditions and that proline, when present at appropriate concentrations in a hyperosmotic environment, can improve both the growth and pullulan yield. Additionally, we performed transcriptomic analysis to elucidate the potential protective mechanisms of proline against high sugar and hyperosmotic stress in *A*. *pullulans*. Transcriptomic analysis helps elucidate the response of an organism to external stressors, identify relevant genes and their expressions, and elucidate the effect of exogenous proline on *A. pullulans* under high-concentration sugar stress conditions. Overall, this study may provide a theoretical foundation for molecular processes in biological fermentation and practical production applications.

## 2. Materials and Methods

### 2.1. Strains and Culture

A mutant *A*. *pullulans* K1 (ATCC 201428) strain with high pullulan production capacity was cultured for 48 h on potato dextrose agar containing 6 g/L potato powder extract, 20 g/L glucose, and 20 g/L agar. A single colony that demonstrated robust growth on the agar plate was selected and transferred to a 250 mL Erlenmeyer flask containing 50 mL of seed medium (6 g/L potato extract powder and 20 g/L glucose) for aerobic culture. The culture was incubated at 28 °C for 20 h under constant shaking at 220 rpm. Thereafter, the cultured solution was stored in a 1.5 mL cryovial containing 50% glycerol and preserved at −80 °C in a refrigerator.

### 2.2. Fermentation Culture

The initial medium for *A*. *pullulans* fermentation was composed of 200 g/L sucrose, 2 g/L yeast extract, 3 g/L NaCl, 0.6 g/L (NH_4_)_2_SO_4_, 6 g/L K_2_HPO_4_, 0.2 g/L MgSO_4_, and 0.06 g/L Fe_2_(SO_4_)_3_. Briefly, the strain was inoculated in the seed culture medium and incubated at 28 °C for 20 h under constant shaking at 220 rpm. Thereafter, the seed culture was transferred to a 500 mL Erlenmeyer flask containing 100 mL of fermentation medium (inoculum volume of 5% [*v*/*v*]) for pullulan production and incubated at 28 °C for 120 h under constant shaking at 220 rpm.

### 2.3. Assessment of High Sugar and Hyperosmotic Stress Tolerance in A. pullulans

*A*. *pullulans* strains were cultured under high sugar and hyperosmotic conditions at the shake flask level, using sucrose as the stress source solute in the fermentation medium to assess their tolerance. Based on the initial fermentation medium, the sucrose concentration of the fermentation medium was adjusted to 80–400 g/L. To more intuitively illustrate the impact of changes in sucrose concentration on osmotic pressure, the corresponding osmotic pressure values were measured using a Fiske Model 210 Freezing Point Osmometer (Advanced, Norwood, MA, USA). Based on the pullulan yield and biomass in the experimental results, sucrose concentrations higher than 120 g/L were defined as high-sugar and hypertonic culture media. Fermentation was performed at 28 °C for 120 h under constant shaking at 220 rpm. Samples were collected at 72, 96, and 120 h of fermentation to determine biomass and pullulan yield under the experimental conditions.

### 2.4. Preparation of Cell-Free Extracts and Determination of Intracellular Amino Acids

Aerobic fermentation was conducted in shake flasks for 72 h, using fermentation media with varying sucrose concentrations. At the midpoint of fermentation (48 h), 5 mL of the fermentation broth was collected and centrifuged at 10,000× *g* for 15 min at 4 °C. After removing the supernatant, the resulting precipitate was washed three times with deionized water, resuspended in 5 mL of pre-cooled phosphoric acid buffer (pH 7.0), and lysed using 50 mg of helicase. Thereafter, the mixture was incubated at 37 °C for 30 min, followed by cell disruption for 15 min to achieve effective cell lysis using an ultrasonic disruptor (SCIENTZ-IID; Ningbo Xinzhi Biotechnology Co., Ltd., Ningbo, China) [17]. The lysate was centrifuged at 8000× *g* for 15 min at 4 °C to remove cell debris, and the resulting supernatant was collected as a cell-free extract [18]. Subsequently, the extract was filtered through a 0.22 μm membrane, followed by the determination of intracellular free amino acid content using an amino acid analyzer (Biochrom30+, Cambridge, UK). The effects of exogenous supplementation with glutamic acid, glycine, and proline on the growth of *A. pullulans* and pullulan production at different sugar concentrations were also explored. At the shake flask level, a high-sugar fermentation medium (200 g/L sucrose) was used as the experimental group, and a low-sugar fermentation medium (100 g/L sucrose) served as the control group. Glutamic acid, glycine, and proline were supplemented at concentrations of 0 mg/L, 100 mg/L, 200 mg/L, 300 mg/L, 400 mg/L, and 500 mg/L, with a fermentation duration of 120 h. Samples were collected for analysis to determine the optimal proline concentration most beneficial for the fermentation process, which guided subsequent experiments.

### 2.5. Determination of Intracellular Glycerol Content

To determine the intracellular glycerol concentration in response to high osmotic pressure, the fermentation broth was collected after 48 h of fermentation with exogenous proline supplementation at different sugar concentrations. The cell-free extract was prepared as described above, and the supernatant was diluted tenfold. A 0.22 μm membrane filter was used, and high-performance liquid chromatography (HPLC) was performed using a Shimadzu system with an Agilent Hi-Plex H column (300 × 7.7 mm; Agilent Technologies Inc., Santa Clara, CA, USA). The mobile phase consisted of a 5 mmol sulfuric acid solution. Intracellular glycerol concentration was measured using a differential refractive index detector (RID-20A), following the detection method outlined by Yang et al. [19].

### 2.6. Scanning Electron Microscopy

Under high-sugar conditions (200 g/L sucrose), exogenous proline (400 mg/L) supplementation was used as a variable. The fermentation broth from the experimental and control groups, fermented for 24 h, was collected and centrifuged at 5000× *g* and 4°C for 20 min to obtain the fungal pellets. The pellets were washed three times with physiological saline, resuspended in pre-cooled 2.5% ammonium chloride, and fixed in dialdehyde for 6 h. The samples were processed according to the method previously described by Yang et al. [20]. Finally, the processed samples were dried at the critical point, sprayed, and coated with gold for observation under the FEI Nova NanoSEM 450 (FEI Company, Hillsboro, OR, USA) scanning electron microscope to acquire images for analysis [21].

### 2.7. Determination of the Activities of Key Enzymes Involved in Pullulan Biosynthesis

Under high-sugar conditions (200 g/L sucrose), exogenous supplementation of proline (400 mg/L) was used as a variable, and aerobic fermentation was conducted at the shake flask level for 120 h. Cell-free extracts were prepared, and enzyme activity was determined. The activities of α-phosphoglucomutase (PGM), UDP–glucosyltransferase (UGT), and UDP–glucose pyrophosphorylase (UGP) were measured using the Yeast PGM ELISA Kit, Yeast UGT ELISA Kit, and Yeast UDPase ELISA Kit (Meiao Biotechnology, Shanghai, China), according to the manufacturer’s instructions. The activities of α-amylase (AMY) and isopullulanase (IPU) were determined using the α-Amylase Activity Assay Kit (Beijing Solarbio Science & Technology Co., Ltd., Beijing, China) and the Isopullulanase Activity Assay Kit (Suzhou Grace Biotechnology Co., Ltd., Suzhou, China), respectively.

### 2.8. Measurement of Biomass, Pullulan Yield, and Molecular Weight in Fermentation Broth

Briefly, 10 mL of fermentation broth was centrifuged at 10,000 rpm for 15 min at 4 °C to obtain the supernatant, which was mixed with 30 mL of pre-cooled absolute ethanol and stored for 12 h. Following precipitation, the mixture was centrifuged at 8000 rpm for 5 min at 4 °C. Thereafter, the precipitated pullulan was washed three times with absolute ethanol to eliminate pigment impurities, dried in an oven at 70 °C until a constant weight was achieved, and weighed to calculate yield [22]. Additionally, the precipitate obtained from the centrifugation of the fermentation broth was washed three times with distilled water, dried in an oven at 70 °C until a constant weight was achieved, and weighed to determine cell biomass [23].

The dried pullulan was pulverized in a grinder, and 1 mg/mL pullulan solution was prepared using ultrapure water. Ten pullulan polysaccharide standards (Sigma, Darm-stadt, Germany) with molecular weights of 342–894 kDa were selected, and 1 mg/mL standard sample solution was prepared [24]. The molecular weight of pullulan, calculated using the sample peak time, was measured using Shimadzu high-performance liquid chromatography according to the detection method previously described by Zhang et al. [25].

### 2.9. Transcriptome Analysis

#### 2.9.1. Sample Preparation

*A*. *pullulans* strains were cultured in a 500 mL baffled Erlenmeyer flask containing 100 mL of medium and fermented at 28 °C under constant shaking at 220 rpm. Two types of fermentation medium were used: an initial fermentation medium and an initial fermentation medium supplemented with 400 mg/L proline. The culture of *A*. *pullulans* grown exclusively in the initial fermentation medium served as the control group (CK) and was divided into three parallel subgroups (CK1, CK2, and CK3). Conversely, cultures grown in the initial fermentation medium with 400 mg/L proline were designated as the experimental group (CP) and comprised three parallel subgroups (CP1, CP2, and CP3). Samples were collected after 24 h of fermentation and centrifuged at 10,000× *g* for 10 min at 4 °C, and the resulting cell pellets were washed three times with sterile water and immediately snap-frozen in liquid nitrogen.

#### 2.9.2. Total RNA Extraction, Library Construction, and Sequencing

The cell pellet was collected, thoroughly ground in liquid nitrogen, and subsequently transferred to a 1.5 mL centrifuge tube. Subsequently, 1 mL of Trizol reagent was added, and the mixture was vortexed to ensure thorough homogenization. The sample was incubated at room temperature for 10 min to allow for complete cell lysis. Total RNA was extracted from the cells using the TRIzol reagent kit (Invitrogen, Carlsbad, CA, USA) following the manufacturer’s guidelines. RNA quality was assessed using an Agilent 2100 Bioanalyzer (Agilent Technologies, Palo Alto, CA, USA) and confirmed using RNase-free agarose gel electrophoresis. Thereafter, eukaryotic mRNA was enriched using oligo (dT) beads, fragmented into shorter segments using a fragmentation buffer, and reverse transcribed to generate cDNA using the NEBNext Ultra RNA Library Prep Kit for Illumina (NEB #7530, New England Biolabs, Ipswich, MA, USA). Purified double-stranded cDNA fragments underwent end repair, were incorporated with an A base, and were ligated to Illumina sequencing adapters. The ligation products were purified using AMPure XP Beads (1.0×) (Beckman Coulter, Brea, CA, USA) and subsequently subjected to polymerase chain reaction (PCR) amplification. Finally, the resulting cDNA library was sequenced on an Illumina Novaseq6000 platform by Gene Denovo Biotechnology Co. (Guangzhou, China).

#### 2.9.3. RNA Sequencing (RNA-seq)

To avoid affecting the subsequent assembly and analysis, low-quality data were filtered to minimize the influence of invalid data. A reference genome index was established to quantify gene abundance and identify differentially expressed genes (DEGs), with the significance threshold set at *p* < 0.05 and |fold change| > 1.5. Gene expression differences between samples were compared and analyzed. Additionally, we performed gene ontology (GO) and Kyoto Encyclopedia of Genes and Genomes (KEGG) pathway enrichment analyses using DEG annotations of the reference genome.

### 2.10. Statistical Analysis

All data are expressed as the mean ± standard deviation (SD) of values obtained from three distinct parallel samples. Significant differences were determined using one-way analysis of variance (ANOVA) with Duncan’s post hoc test. Statistical significance was set at *p* < 0.05. All analyses were performed using SPSS 27.0 (IBM, New York, NY, USA).

## 3. Results

### 3.1. A. pullulans Growth and Pullulan Synthesis Under High Sugar and Hyperosmotic Stress

**Figure 1:**
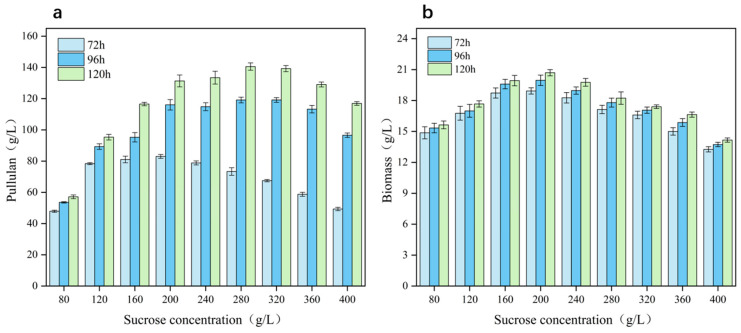
Effect of different sugar concentrations on pullulan production and *Aureobasidium pullulans* growth: Pullulan yield (**a**); cell biomass (**b**).

To evaluate the effects of environmental stress on *A*. *pullulans* growth and pullulan production, *A*. *pullulans* was fermented under high sugar and hyperosmotic conditions. Flask fermentation experiments were performed using different sucrose concentrations (80–400 g/L). Notably, the osmotic pressure of the fermentation system increased from 441.7 ± 5.7 to 1965 ± 16.7 mosmol/kg with increasing sucrose concentration (the results of the osmolality measurement are shown in Supplementary Figure S1). Additionally, pullulan yield and fungal biomass exhibited an initial increase with increasing sucrose concentration, followed by a decrease (Figure 1). During the middle stage of fermentation (at 72 h), pullulan yield and biomass peaked at sucrose concentrations of 120 g/L, followed by a decrease at concentrations higher than 200 g/L. Importantly, pullulan yield at a sucrose concentration of 400 g/L was only 49.37 g/L (12.34%), representing a decrease of 40.54% compared with the highest yield of 83.03 g/L. In the late and final stages of fermentation (96 h and 120 h), pullulan yield peaked (140.57 g/L; 50.2%) at a sucrose concentration of 280 g/L (production intensity 1.17 g/L/h), which may be owing to abundant carbon source supply in the later stages of fermentation. Therefore, an appropriate increase in sugar concentration can improve polysaccharide and fungal biomass yield. However, if the sugar concentration increases further, the osmotic pressure will stress the fungus, thereby inhibiting pullulan biosynthesis.

### 3.2. Effect of High Sugar and Hyperosmotic Stress on Intracellular Amino Acid Content in A. pullulans

**Figure 2:**
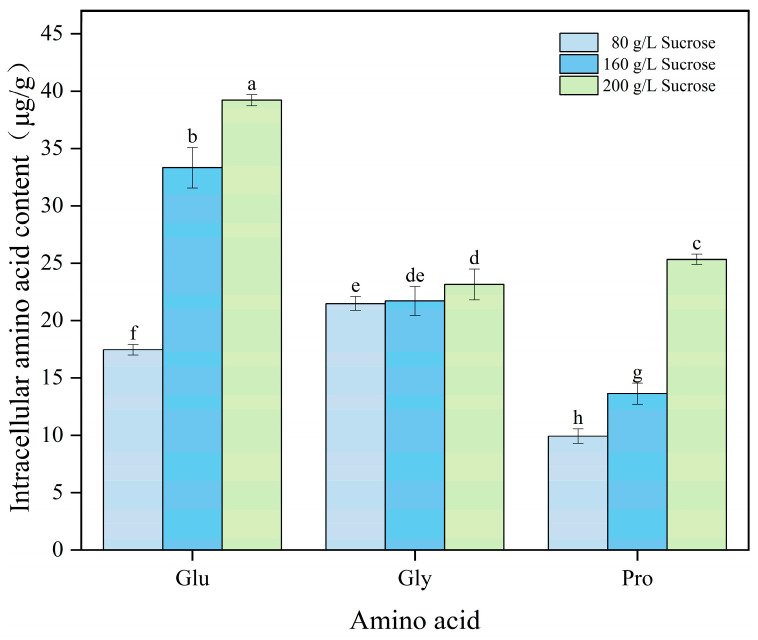
Changes in the contents of glutamic acid, glycine, and proline under different sucrose concentrations were evaluated. Significant differences (*p* < 0.05) were determined using a one-way analysis of variance with Duncan’s test and represented using different letters.

In many microorganisms, amino acids and their derivatives serve as intracellular compatible solutes that help resist environmental stress. Glycine, proline, and glutamic acid are frequently used as osmoprotectants to mitigate hypertonic stress in fermentation systems [26]. To investigate the effects of varying sucrose concentration on intracellular amino acid composition and content in *A*. *pullulans*, we measured the intracellular free amino acid content during hyperosmotic stress using an amino acid analyzer. The glutamic acid, glycine, and proline contents of *A*. *pullulans* increased to varying degrees with increasing sucrose concentration in the culture environment (Figure 2). Compared with those in the low-sucrose group (80 g/L), glutamic acid, glycine, and proline contents increased by 55.47%, 7.18%, and 60.83%, respectively, in the high-sucrose group (200 g/L). These three amino acids accumulated to varying degrees as osmoprotectants in stressed cells compared with those in cells under normal osmotic pressure.

### 3.3. Protective Effects of Amino Acids Against Hyperosmotic Stress in A. pullulans

**Figure 3:**
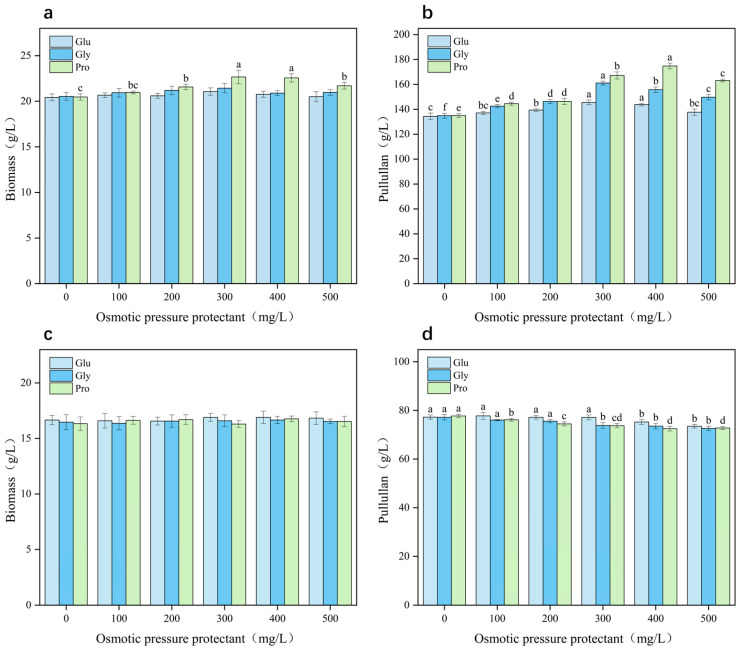
Effects of exogenous supplementation of glutamic acid, glycine, and proline on biomass (**a**) and pullulan yield (**b**) under high sugar conditions (200 g/L) and on biomass (**c**) and pullulan yield (**d**) under low sugar conditions (100 g/L). Significant differences (*p* < 0.05) were determined using a one-way analysis of variance with Duncan’s test and represented using different letters.

Considering that glutamic acid, glycine, and proline contents increased in *A*. *pullulans* with increasing osmotic pressure in the fermentation system, it could be speculated that these amino acids are necessary to counteract the inhibitory effects of hypertonic stress on cell growth. Thus, we investigated whether the exogenous addition of amino acids contributes to the biosynthesis of pullulan and the growth of *A. pullulans*. Treatment with the amino acids significantly enhanced the growth of *A*. *pullulans* under hyperosmotic stress (Figure 3a,b). The maximum biomass achieved was 22.67 ± 0.74 g/L, representing a 10.75% increase compared with that in the control group. Additionally, the yield of pullulan reached its peak at 174.8 ± 2.31 g/L, with a yield of 0.874 g/L (production intensity, 1.46 g/L/h), at a proline concentration of 400 mg/L, representing a 30.06% increase compared with that in the control group. Under low sugar conditions, adding different concentrations of amino acids had no effect on fungal growth (Figure 3c,d). Additionally, as the concentration of amino acids increased, the yield of pullulan decreased slightly. Therefore, proline was effective in promoting *A. pullulans* growth and pullulan production under high sugar and hyperosmotic conditions. The exogenous addition of 400 mg/L proline protected against stress associated with 200 g/L sucrose and optimized pullulan biosynthesis.

### 3.4. A. pullulans Transports Exogenous Amino Acids to Resist High Sugar Stress

**Figure 4:**
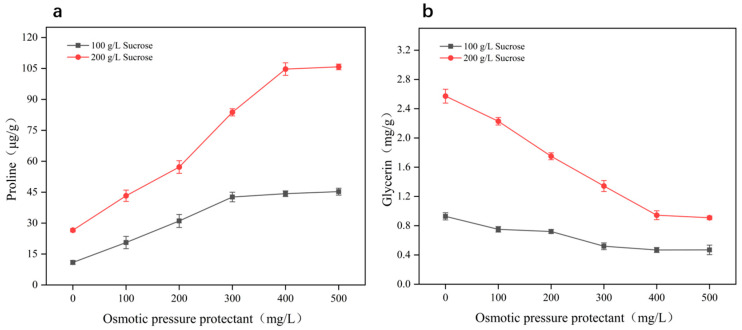
Effect of exogenous proline on proline (**a**) and glycerol (**b**) concentration in *Aureobasidium pullulans* cells at different sucrose concentrations (100 and 200 g/L).

We measured changes in intracellular proline concentration and glycerol after 48 h of fermentation with exogenous proline supplementation under different sucrose concentrations (100 and 200 g/L) to determine whether *A. pullulans* can transport exogenous amino acids from the external environment into the cell to balance osmotic pressure. Glycerol, a key compatible solute, plays a crucial role in cells’ response to osmotic pressure changes, and its concentration variations reflect the adaptation of *A. pullulans* to high-sugar stress. By monitoring the changes in the concentrations of these substances, we can further investigate the regulatory role of proline transport in maintaining intracellular osmotic balance in a hyperglycemic and hypertonic environment. The results are shown in Figure 4. Under the same sucrose concentration, increasing the amount of exogenous proline led to a significant increase in intracellular proline content, while intracellular glycerol concentration decreased significantly. This trend was more pronounced at a sucrose concentration of 200 g/L. Specifically, the intracellular proline content of *A. pullulans* increased by up to 298.34%, while glycerol content decreased by 64.66% (the results of intracellular glycerol content HPLC analysis are shown in Supplementary Figure S2), indicating that a large amount of exogenous proline was transported into the cells and utilized to counteract high glucose and hyperosmotic stress. As osmotic pressure decreased, intracellular glycerol accumulation was reduced. Furthermore, when the same concentration of proline was supplemented, the intracellular proline and glycerol contents in a high-sugar environment were significantly higher than in a low-sugar environment, suggesting that the high-sugar condition promotes the transport of more proline into the cells, where it accumulates as a compatible solute to protect cell growth and metabolism.

### 3.5. Effect of Exogenous Proline on A. pullulans Cell Surface Morphology

**Figure 5:**
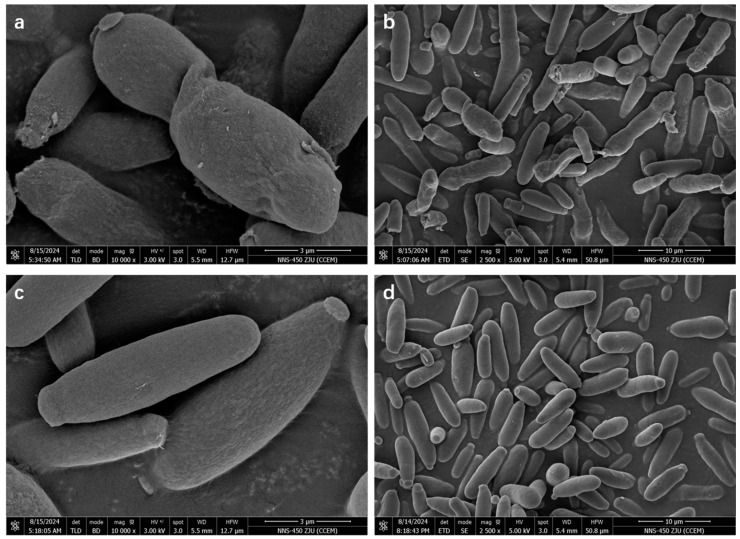
Comparative analysis of scanning electron microscopy images of the experimental and control groups in the absence of proline: (**a**) Control cells, magnification: 10,000×. (**b**) Control cells, magnification: 2500×. (**c**) Cells in the experimental group, magnification: 10,000×. (**d**) Cells in the experimental group, magnification: 2500×.

Figure 5 shows the cell surface morphology of *A. pullulans* following treatment with proline. In the control group (Figure 5a,b), cell surfaces were deformed, wrinkled, or even damaged under high sugar stress. By contrast, the cell structure was intact, and the cell surface was smooth in the experimental group following proline treatment (Figure 5c,d). Therefore, proline may serve as a compatible solute, and *A*. *pullulans* can transport exogenous proline from the culture medium to the intracellular environment to maintain cell membrane integrity and improve resistance to hypertonic stress.

### 3.6. Effect of Exogenous Addition of Proline on the Activities of Key Enzymes

**Figure 6:**
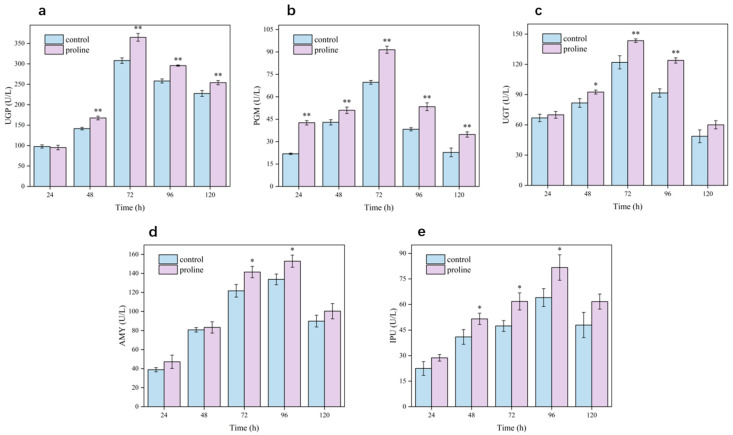
Activities of pullulan biosynthetic and degrading enzymes in the experimental and control groups in the presence or absence of proline at different fermentation stages. UGP: UDP–glucose pyrophosphorylase (**a**); PGM: α-phosphoglucomutase (**b**); UGT: UDP–glucosyltransferase (**c**); AMY: α-amylase (**d**); IPU: isopullulanase (**e**). Asterisks indicate the level of significance using the Student’s *t*-test (* *p*  <  0.05; ** *p*  <  0.01) in comparison to the control without proline addition.

Key enzymes involved in the pullulan biosynthetic pathway during *A*. *pullulans* fermentation include UGP, PGM, and UGT. Additionally, AMY and IPU are crucial enzymes that degrade pullulan and affect its molecular weight [27,28]. Therefore, we examined the effects of exogenous proline on the activities of pullulan biosynthetic and degrading enzymes at various stages of the fermentation process. The control and experimental groups exhibited slow cell growth and low biosynthetic enzyme activity under a high-sugar environment during the first 24 h of fermentation (Figure 6a–c). However, the activities of pullulan biosynthetic enzymes (UGP, PGM, and UGT) increased with increasing duration of fermentation, peaking at 72 h, followed by a decrease in the later stages of fermentation (96 and 120 h). This decline may be attributed to the increased viscosity of the fermentation broth, depletion of nutrients, and inhibition of cell growth and metabolism during the later stages, which ultimately affected the pullulan synthesis pathway. Proline supplementation increased the activities of pullulan biosynthetic enzymes to varying degrees in the experimental group at each fermentation stage. During the early fermentation stage (24 h), PGM activity was significantly higher in the proline group than in the control group; however, there were no significant differences in the activities of the other enzymes between the groups. During the middle and late stages (72 and 96 h), UGP, PGM, and UGT activities were significantly higher in the proline group than in the control group. Furthermore, the increase in the activities of these key enzymes enhanced pullulan synthesis. Additionally, proline addition significantly increased the activities of the pullulan-degrading enzymes AMY and IPU during the middle and late stages of fermentation (72 and 96 h; Figure 6d,e). Collectively, these findings indicate that the addition of an appropriate amount of proline to the fermentation medium enhanced the activities of pullulan biosynthetic enzymes, thereby improving pullulan yield and production intensity. Additionally, the proline-induced upregulation of pullulan synthesis enhanced the activity of degrading enzymes, contributing to an increase in pullulan production but a decrease in molecular weight (the results of HPLC analysis are shown in Supplementary Figure S3). To further elucidate the mechanism by which proline affects *A*. *pullulans*, we conducted the transcriptomic analysis.

### 3.7. Effect of Exogenous Proline on A. pullulans Under High Sugar Stress Conditions at the Transcriptome Level

Exogenous proline mitigated the high sugar stress response in *A*. *pullulans* and significantly enhanced pullulan biosynthesis. To elucidate the effect of exogenous proline on *A. pullulans* under high sugar stress conditions, we performed transcriptomic analysis of cells in the control and experimental groups following supplementation with 400 mg/L proline.

#### 3.7.1. RNA-seq Results and Quality Control

Table 1 shows details of RNA-seq reads for each sample. After quality control filtering, the six samples yielded 34,652,489,808 clean reads, with Q20 and Q30 values of >98% and >94%, respectively. The high quality of the sequencing data ensured the accuracy of subsequent analyses.

#### 3.7.2. Statistical Analysis of DEGs

**Figure 7:**
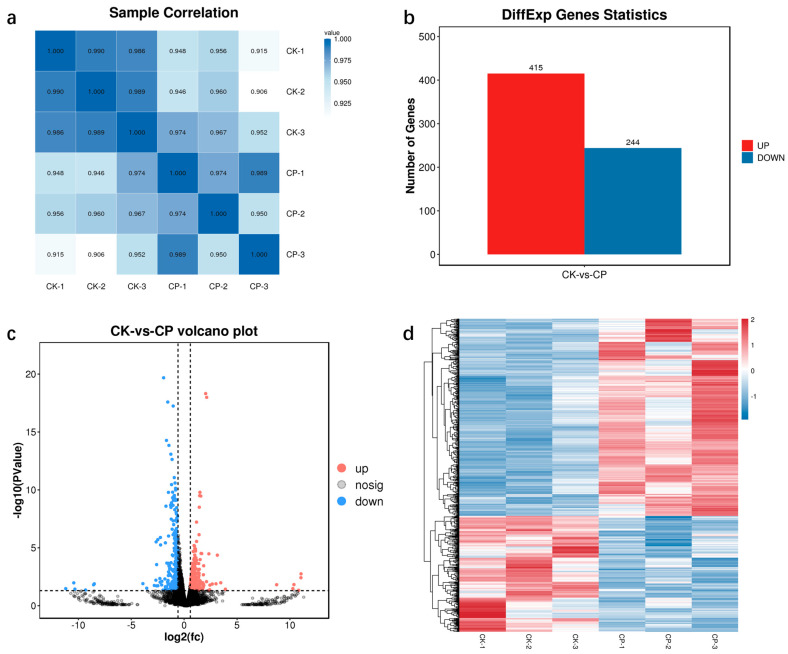
Sample correlation heat map (**a**). Statistics of differentially expressed genes (DEGs) (**b**). Volcano plot (**c**) and heatmap (**d**) showing gene expression patterns in the control (CK) and proline (CP) groups. CK: *Aureobasidium pullulans* cultivated for 24 h in the initial fermentation medium; CP: *A*. *pullulans* cultivated for 24 h in the fermentation medium containing proline. In the volcano plot of differential genes, each point represents a gene, with red representing upregulation and blue representing downregulation. In the differential comparison clustering heat map, the expression levels of genes in different samples are represented by different colors.

Correlation analysis was performed to assess the reliability of the transcriptome data. A correlation coefficient close to 1 indicates a high similarity in gene expression patterns among the samples. The coefficient values for each sample ranged from 0.906 to 1, indicating strong repeatability within the same group, significant differences between groups, and high reliability of data (Figure 7a). Additionally, differential expression analysis was performed to identify DEGs between the proline and control groups. In total, 659 DEGs were identified, among which 415 were significantly upregulated, and 244 were significantly downregulated (Figure 7b). Genetic differences between the comparison groups were visualized using a volcano plot (Figure 7c). Moreover, the expression patterns of the genes were visualized using a heatmap (Figure 7d). Genes exhibiting similar expression patterns may share common functions or be involved in related metabolic and signaling pathways.

#### 3.7.3. GO Functional Enrichment Analysis of DEGs

**Figure 8:**
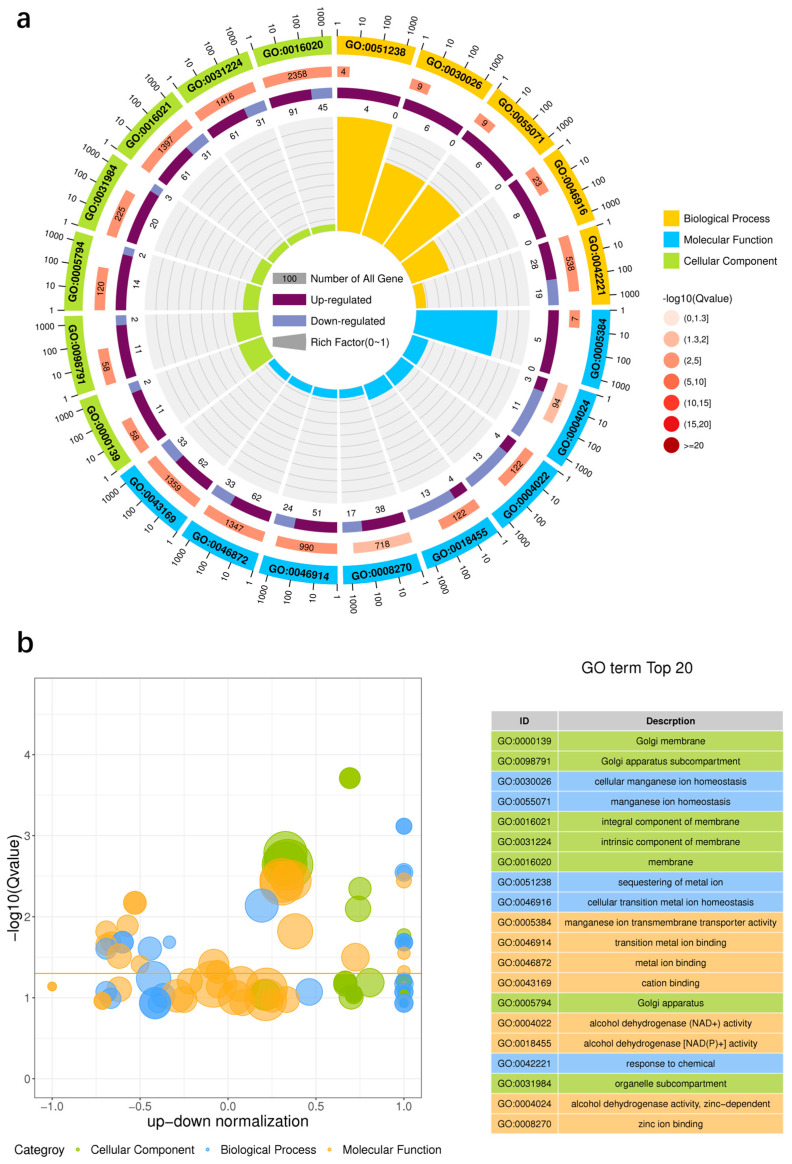

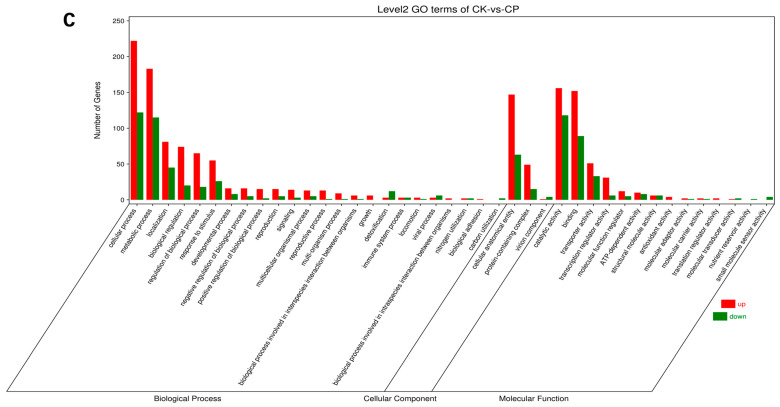
Gene ontology (GO) enrichment circle diagram (**a**): The first circle represents the top 20 enriched GO terms, the second circle represents the number and Q value of the GO term in the background of differential genes, and the third circle represents the proportion of upregulated and downregulated DEGs. GO enrichment difference bubble chart (**b**): The ordinate is -log10 (Q value), and the abscissa is the z-score value. GO enrichment classification histogram (**c**): abscissa is the secondary GO term, ordinate is the number of differential genes in the term, and different colors represent different types of GO terms.

To explore the mechanisms of proline, we performed GO functional annotation analysis of DEGs between the proline and control groups. Figure 8a,b represent the GO enrichment circle diagram and enrichment difference bubble diagram of the DEGs, respectively. The DEGs were mainly enriched in eight molecular functions, seven cellular components, and five biological processes. Additionally, the biological processes (Figure 8c) included cellular processes (222 upregulated and 122 downregulated), metabolic processes (183 upregulated and 115 downregulated), localization (81 upregulated and 45 downregulated), and biological regulation (74 upregulated and 20 downregulated). Moreover, the cellular components included cellular anatomical entities (147 upregulated and 63 downregulated) and protein-containing complexes (49 upregulated and 15 downregulated). Furthermore, the significantly enriched molecular functions included catalytic activity (156 upregulated and 118 downregulated), binding (152 upregulated and 89 downregulated), and transporter activity (51 upregulated and 33 downregulated). The significant upregulation of these DEGs is likely to facilitate cell growth, promoting the adaptation of *A*. *pullulans* to external environmental stress. Overall, this finding is crucial for understanding the regulatory mechanisms underlying stress response in *A*. *pullulans*.

### 3.8. KEGG Enrichment Analysis

**Figure 9:**
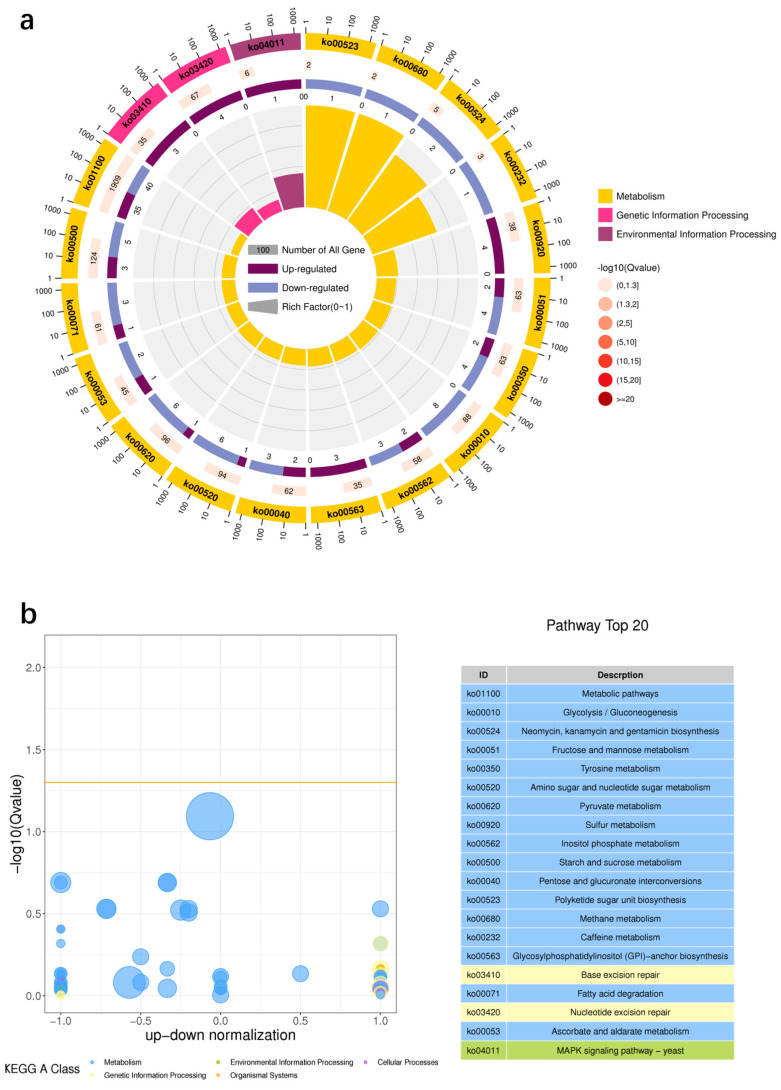

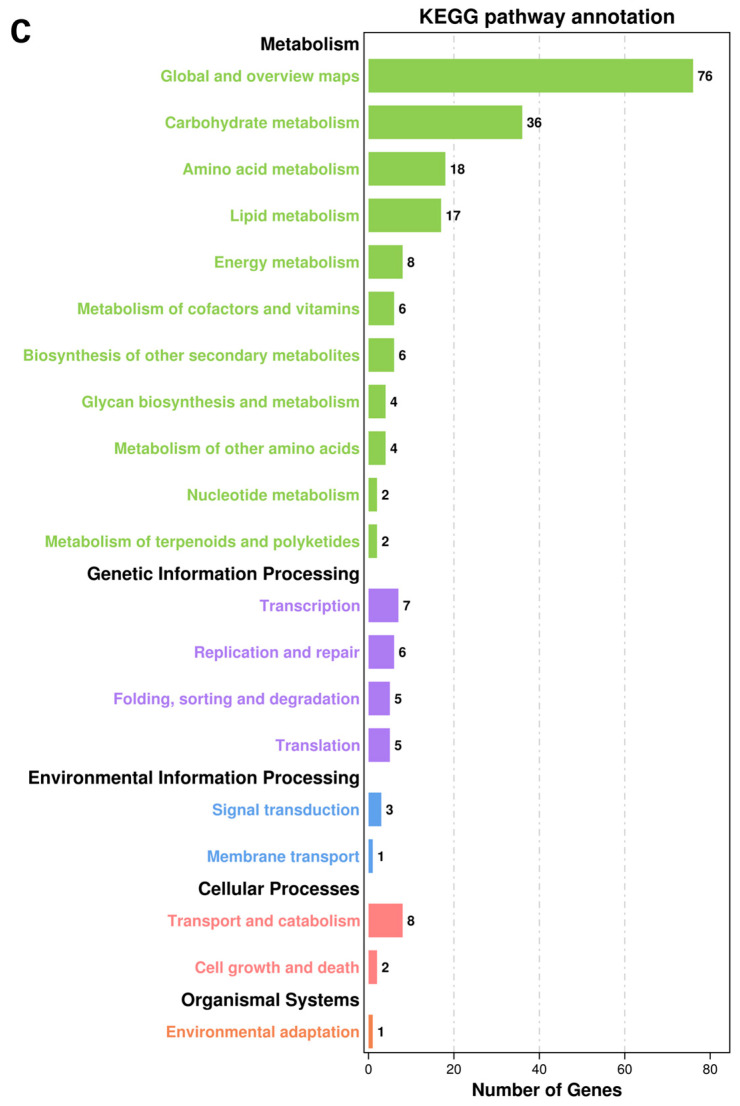
Kyoto Encyclopedia of Genes and Genomes (KEGG) enrichment circle diagram (**a**): The first circle represents the top 20 enriched pathways, the second circle represents the number and Q value of the pathway, and the third circle represents the proportion of upregulated and downregulated DEGs. KEGG enrichment difference bubble chart (**b**): The ordinate is −log10 (Q value), and the abscissa is the z-score value. KEGG enrichment secondary classification histogram (**c**): the top 20 KEGG enriched pathways.

To elucidate the metabolic pathways by which proline exerts its protective effect against high sugar stress in *A*. *pullulans*, we performed KEGG metabolic pathway enrichment analysis of DEGs between the proline and control groups. In total, 109 DEGs were enriched in various KEGG pathways (Figure 9). Compared with those in the control group, DEGs in the experimental group were associated with 84 pathways, among which 11 were significantly enriched (*p* < 0.05). In total, 68.8% of the DEGs were significantly enriched in metabolic pathways. Specifically, the 20 most enriched metabolic pathways belonged to three categories: metabolism, genetic information processing, and environmental information processing. Pathways that primarily influenced growth, reproduction, energy metabolism, antagonism, and signal transduction in *A*. *pullulans* included metabolic pathways (Ko01100); glycolysis/gluconeogenesis (Ko00010); fructose and mannose metabolism (Ko00051); amino sugar and nucleotide sugar metabolism (Ko00520); pyruvate metabolism (Ko00620); starch and sucrose metabolism (Ko00500); sulfur metabolism (Ko00920); and the MAPK signaling pathway—yeast (Ko04011).

The upregulated DEGs were predominantly associated with functional categories, including metabolic pathways (Ko01100); sulfur metabolism (Ko00920); glycosylphosphatidylinositol (GPI)—anchor biosynthesis (Ko00563); base excision repair (Ko03410); nucleotide excision repair (Ko03420); and the MAPK signaling pathway in yeast (Ko04011). Conversely, the downregulated DEGs were mainly associated with functional categories, including glycolysis/gluconeogenesis (Ko00010), amino sugar and nucleotide sugar metabolism (Ko00520), and pyruvate metabolism (Ko00620).

KEGG pathway annotations encompassed five categories: metabolism, genetic information processing, environmental information processing, cellular processes, and biological processes. Exogenous proline treatment significantly influenced key metabolic pathways in *A*. *pullulans*, including carbohydrate, amino acid, sulfur, lipid, and energy metabolism. Additionally, proline treatment affected transcription, replication, repair, folding, selection, degradation, translation, signaling, and transport.

## 4. Discussion

Pullulan, which is primarily utilized in the food and cosmetic industries, is synthesized and secreted by various strains of Aureobasidium spp. [29]. During industrial production, high sugar concentrations inhibit pullulan biosynthesis. Typically, sugar concentrations exceeding 5% of the carbon source adversely affect pullulan synthesis and increase production costs, necessitating an extension of germination time. Therefore, the tolerance of *Aureobasidium* spp. to high sugar and hyperosmotic conditions during fermentation presents an urgent challenge requiring resolution [30,31]. In this study, we examined the protective effect and mechanisms of proline against high sugar and hyperosmotic stress in *A*. *pullulans* to enhance pullulan yield and reduce costs. Additionally, we conducted a comprehensive investigation of the impact of high sugar and hyperosmotic environments on *A*. *pullulans* growth and pullulan biosynthesis. Our findings indicate that an increased substrate concentration results in elevated osmotic pressure, which inhibits fungal growth and metabolism. Notably, an appropriate increase in sugar concentration can improve polysaccharide and fungal biomass yield. However, the osmotic pressure of the fermentation system increased gradually with increasing substrate concentration in this study. Osmotic pressure is an environmental stress factor that inhibits the growth and metabolism of *A*. *pullulans*. Improving hyperosmotic stress resistance in *A*. *pullulans* is an important research direction for increasing pullulan yield and reducing industrial production costs.

Microorganisms accumulate compatible solutes in hypertonic environments to manage stress, and amino acids and their derivatives play crucial roles in this process [32,33]. Quantitative analysis confirmed the accumulation of intracellular amino acids in *A*. *pullulans* under hypertonic conditions. Furthermore, it was found that adding an appropriate amount of proline to a high-sugar fermentation medium significantly increased both biomass and pullulan production, while no significant changes were observed in low-sugar cultures. To explore the possible reasons for the increased pullulan production, we measured the changes in intracellular proline concentration and glycerol following the addition of exogenous proline. Previous studies have shown that glycerol accumulates in large quantities under high external osmotic pressure [34]. In our experiment, the addition of exogenous proline significantly increased intracellular proline levels while intracellular glycerol content decreased notably. This further supports the role of proline as an osmoprotectant. Due to the limited amount of amino acids added in this experiment, the effect of exogenous amino acids as nutritional components in promoting cell growth was not pronounced. Particularly under high sugar and hyperosmotic stress conditions, cells synthesize large amounts of proline as an osmoprotectant. However, the concentration of proline synthesized endogenously by the cells is low and insufficient to fully counteract high sugar and hyperosmotic stress. Therefore, the addition of exogenous proline allows *A*. *pullulans* to transport and accumulate within the cells, effectively mitigating the osmotic stress caused by the high-sugar environment and maintaining normal cellular metabolism and growth. This finding offers new insights and strategies for improving pullulan production efficiency in high-sugar environments. To elucidate the physiological mechanism by which exogenous proline improves pullulan synthesis, we examined cell morphology and the activities of key enzymes involved in pullulan biosynthesis and degradation in the experimental and control groups. In the experimental group, the cell surface appeared smooth with intact morphology following proline addition. Additionally, proline treatment enhanced the activity of key enzymes (UGP, PGM, and UGT) involved in pullulan synthesis. Collectively, these findings indicate that proline effectively alleviates high sugar and hyperosmotic stress, thereby improving cell growth and metabolic processes and increasing pullulan synthesis.

A high-sugar environment triggers a series of stress responses in *A*. *pullulans*, affecting cell growth, energy metabolism, transcription, and translation [35,36]. Considering the accuracy and efficiency of RNA-seq technology [37], we performed transcriptomic analysis to elucidate the mechanism of stress resistance in *A*. *pullulans*. We comprehensively investigated key genes and associated metabolic pathways associated with high sugar stress using RNA-seq, differential expression analysis, and GO and KEGG enrichment analyses. Proline upregulated genes are associated with sulfur metabolism, base excision repair, nucleotide excision repair, and the MAPK signaling pathway, and downregulated genes are related to glycolysis and the acetone metabolism pathway. High glucose stress often enhances the expression of genes associated with glycolysis and the pentose phosphate pathway, which are crucial for the breakdown of glucose to generate ATP [38]. However, proline treatment downregulated DEGs in glycolysis/gluconeogenesis and pyruvate metabolism pathways during the early stage of fermentation; our study indicates that the addition of an appropriate amount of proline enhances the resistance of microorganisms to hypertonic conditions. This explains why the proline-induced mitigation of stress response in *A*. *pullulans* was associated with the downregulation of genes associated with energy metabolism. Additionally, DEGs in pathways related to genetic and environmental information processing were significantly upregulated, suggesting that the enhancement of cell growth and stress resistance in the early stages of fermentation effectively protects cells from environmental stress damage.

Under environmental stress conditions, the genetic material of microorganisms may sustain varying degrees of damage, leading to reduced DNA synthesis and slower growth rates [39,40]. In the present study, high sugar stress inhibited the normal proliferation and growth of *A*. *pullulans*. Proline addition to the culture medium significantly upregulated genes associated with DNA synthesis and repair, including formamidopyrimidine-DNA glycosylase (*fpg1*), DNA ligase 1 (*adl1*), thymine-DNA glycosylase (*thp1*), transcription initiation factor TFIIH subunit 4 (*tfb2*), replication factor C subunit 3/5 (*b14d6.460*), and DNA repair protein complementing XP-A cells (*rhp14*). Proline treatment enhanced intracellular DNA synthesis in *A*. *pullulans*, indicating that proline alleviates high sugar stress. This finding was consistent with the observed increase in biomass in the experimental group following proline addition during fermentation.

Sulfur metabolism is a crucial metabolic activity in microorganisms that affects stress tolerance [41]. In the present study, proline treatment upregulated genes associated with the sulfur metabolism pathway, including sulfite oxidase (*suox*) and phosphoadenosine phosphosulfate reductase (*sA*), potentially leading to alterations in intracellular sulfur content. Modulating the concentration of sulfur-containing compounds, such as hydrogen sulfide, cysteine, and glutathione, protects cells from various stressors during fermentation [42,43]. Additionally, DEGs related to sulfur metabolism pathways may positively affect stress resistance in *A*. *pullulans*.

This study has certain limitations. Considering that only samples from the initial 24 h of fermentation were selected for transcriptome analysis, the relationship between proline production mechanism and fermentation duration remains inconclusive. During the fermentation process, changes in the biosynthetic and metabolic pathways of microorganisms might occur at different time points, resulting in different gene expression and protein synthesis. To overcome these limitations, additional time points covering the entire fermentation process can be selected for sampling. In this way, more comprehensive gene expression data can be obtained to further analyze the relationship between proline and pullulan biosynthetic pathways at different time points. In the future, multi-omics analysis should be performed at different stages of fermentation, and multi-omics technology such as proteomics and metabolomics should be used to analyze protein expression levels and metabolite changes to reveal the potential role of proline in improving pullulan production [44,45]. This will help optimize the production process, increase the yield of pullulan, and provide an important theoretical basis for regulating pullulan fermentation.

## 5. Conclusions

Exogenous proline supplementation enhances *A*. *pullulans* growth and pullulan biosynthesis in a high-sugar and hyperosmotic environment. Proline supplementation (400 mg/L) increased biomass and pullulan yield by 10.75% and 30.6% (174.8 g/L), respectively, compared with those in the control group. Additionally, we elucidated the molecular mechanisms by which proline protects *A*. *pullulans* against high sugar stress using physiological, biochemical, and transcriptomic analyses. Collectively, our findings offer valuable insights into the application of proline to mitigate hyperosmotic stress responses in *A*. *pullulans*. Proline is an inexpensive and readily available material that can be utilized in the industrial production of pullulan to enhance the polysaccharide yield and reduce production costs.

## Figures and Tables

**Table 1:** Base quality analysis statistics.

Sample	Raw Data (bp)	Clean Data (bp)	Q20 (%)	Q30 (%)	N (%)	GC (%)
CK-1	5,988,940,500	5,950,751,278	5,853,778,416 (98.37%)	5,653,430,973 (95.00%)	91,353 (0.00%)	3,118,360,617 (52.40%)
CK-2	6,232,372,500	6,197,207,194	6,093,540,111 (98.33%)	5,885,423,028 (94.97%)	102,951 (0.00%)	3,257,455,686 (52.56%)
CK-3	5,909,783,400	5,855,383,584	5,758,623,630 (98.35%)	5,551,683,529 (94.81%)	165,982 (0.00%)	3,066,103,894 (52.36%)
CP-1	5,752,149,000	5,700,962,758	5,621,846,659 (98.61%)	5,451,164,121 (95.62%)	103,635 (0.00%)	2,961,035,665 (51.94%)
CP-2	5,523,115,500	5,484,849,961	5,393,385,771 (98.33%)	5,210,109,566 (94.99%)	92,173 (0.00%)	2,842,443,729 (51.82%)
CP-3	5,508,067,500	5,463,335,033	5,358,162,917 (98.07%)	5,155,132,240 (94.36%)	92,332 (0.00%)	2,798,609,660 (51.23%)

## Data Availability

The original contributions presented in the study are included in the article/Supplementary Materials, further inquiries can be directed to the corresponding author.

## Supplementary Materials

The following supporting information can be downloaded at: https://www.mdpi.com/article/10.3390/microorganisms12122657/s1, Figure S1. Measurement of the osmotic pressure in the fermentation system corresponding to the sucrose concentration in the culture medium. Figure S2. The results of HPLC analysis of standard glycerol concentration. Figure S3: The results of HPLC analysis of the product pullulan between the control group and the experimental group with added proline.
