# Supplementary material for: Proline Improves Pullulan Biosynthesis Under High Sugar Stress Condition
Source: Microorganisms. 2024 Dec 21;12(12):2657. doi: 10.3390/microorganisms12122657 (PMC11728471; doi:10.3390/microorganisms12122657)
Supplement: Supplementary file 1 [file microorganisms-12-02657-s001.zip › microorganisms-3301264-supplementary.pdf]

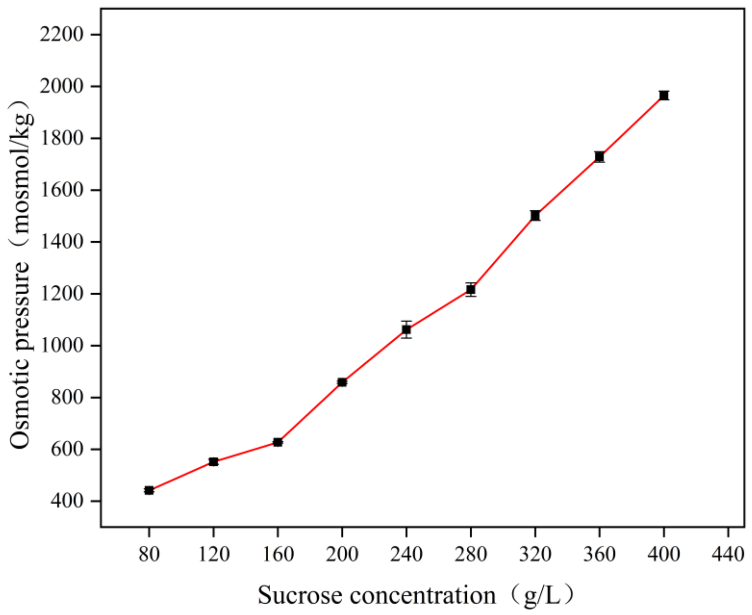

Figure S1. Measurement of the osmotic pressure in the fermentation system corresponding to the sucrose concentration in the culture medium.

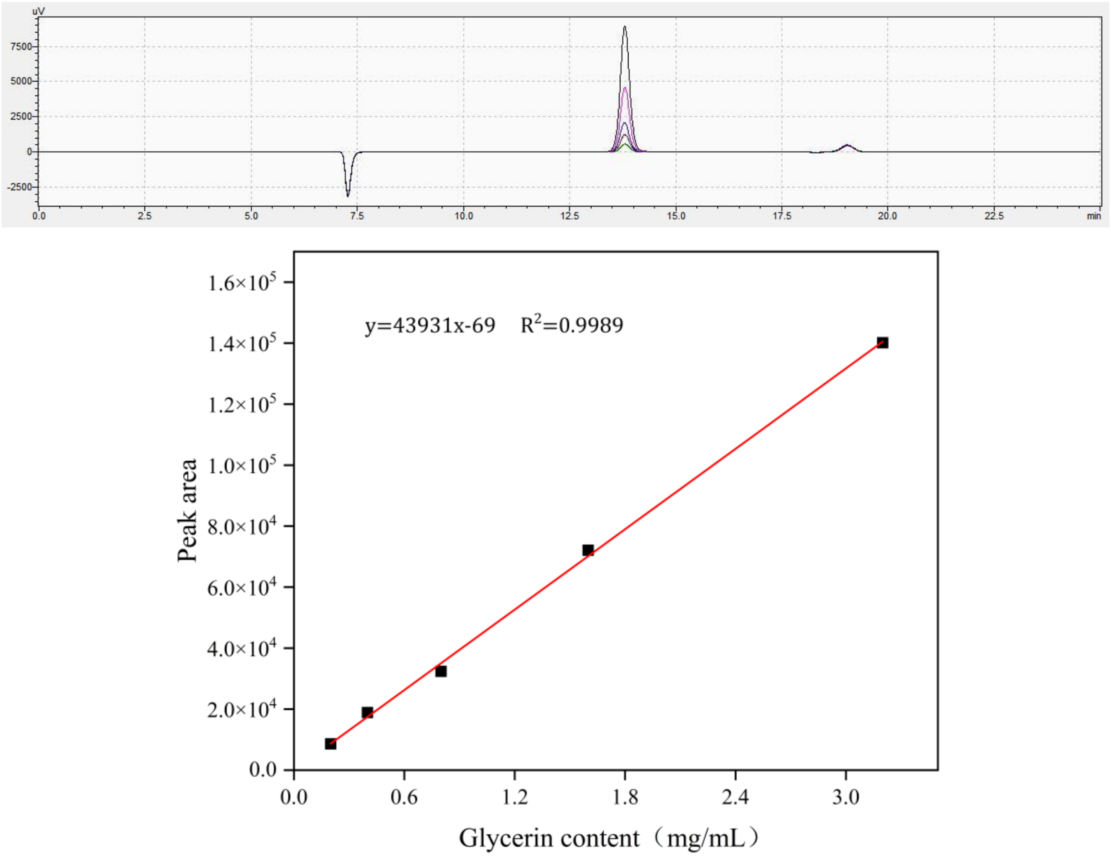

Figure S2. The results of HPLC analysis of standard glycerol concentration.

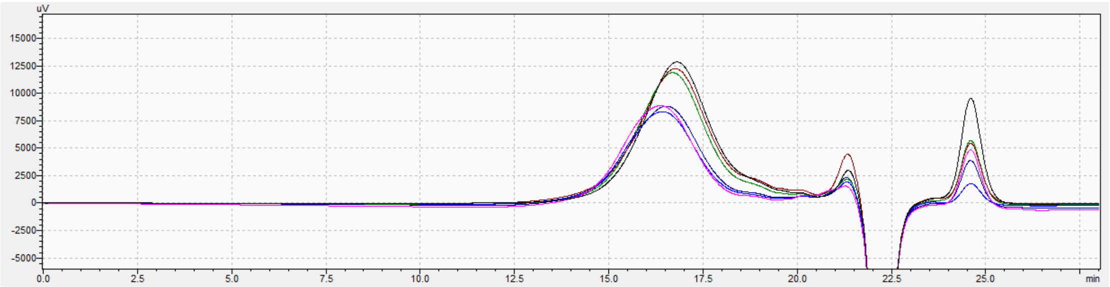

Figure S3. The results of HPLC analysis of the product pullulan between the control group and the experimental group with added proline.
